# Multiple Placental Abruptions With Fetal Loss: A Case Report and Review of the Literature

**DOI:** 10.7759/cureus.91251

**Published:** 2025-08-29

**Authors:** Jacqueline Figueroa, Olivia Berutti, Nicholas Pulido, Antonio Maceri, Nam Le, Aamir Shakil, Talha Khan, Ata Atogho

**Affiliations:** 1 Medicine, American University of the Caribbean Medical School, Cupecoy, MAF; 2 Medicine, Ross University School of Medicine, Bridgetown, BRB; 3 Obstetrics and Gynaecology, Jackson Health System, Miami, USA

**Keywords:** evidence-based clinical guidelines, fetal loss, literature review of disease, placental abruption, review of literature

## Abstract

The risk for negative outcomes in a pregnancy increases substantially when the placenta disconnects from the endometrial lining too soon before birth. This serious complication in obstetrics is known as placental abruption. Here, we describe a 39-year-old G5P4003 patient at 32 weeks of gestation who presented to the emergency department complaining of absent fetal movement and abdominal pain. Her medical history was notable for multiple high-risk factors, including a past placental abruption with fetal demise, requiring cesarean delivery at 39 weeks. She reported taking her antihypertensive medications consistently but admitted she had missed her most recent prenatal appointment. The emergency ultrasound confirmed a repeat abruption.

The patient in this case had many risk factors for placental abruption, including gestational hypertension, preeclampsia and eclampsia, and advanced maternal age, as well as a previous pregnancy resulting in an abruption with fetal demise. High-risk patients such as these need to work closely with their medical team and follow the recommended guidelines to have the best chance of a healthy delivery. Still, this case exposes weaknesses in the current protocols for delivery timing and monitoring of high-risk pregnancies, as well as highlighting the importance of thorough patient education. Further research is needed to develop improved evidence-based recommendations for the best management practices, delivery schedules, and risk stratification in pregnancies at high risk of abruption.

## Introduction

In pregnancy, the placenta attaches itself to the uterine endometrial epithelium and grows into a special organ that maintains embryonic development by eliminating waste products from metabolism and delivering essential nutrients [1]. When this vital exchange system is prematurely separated, the mother can experience severe bleeding, and the fetus becomes endangered [2], leading to possible morbidity and demise. Placental abruption complicates 0.6% and 1.2% of pregnancies [3,4], and once a patient experiences one abruption, they now have a higher risk of experiencing another [5]. When paired with other risk factors for abruption, such as advanced maternal age, hypertension, and preeclampsia or eclampsia, the risk increases even further.

Despite the established risk factors, there is limited evidence-based consensus on the suitable policies for handling these high-risk pregnancies. By presenting a patient with multiple risk factors who experienced recurrent placental abruption that led to intrauterine fetal demise, this case adds to the literature by contributing in four ways: (i) by demonstrating how risk factors can compound to influence adverse outcomes; (ii) by highlighting inadequacies in the frequency and timing of current monitoring protocols; (iii) by emphasizing the need for improved patient education regarding the risks of recurrence and warning signs that require immediate attention; and (iv) by encouraging more training for medical staff to recognize high-risk patients and warning signs for faster intervention. By pursuing this case, we aim to bring attention to the risk for recurrent placental abruption in pregnancies with compounding risk factors and emphasize the critical need for development of evidence-based management protocols.

## Case presentation

At 32 weeks’ gestation, a 39-year-old G5P4003 African American woman arrived at the emergency department complaining of abdominal pain and no fetal movement. Her medical history included gestational hypertension, a past placental abruption with fetal demise requiring cesarean delivery at 39 weeks, eclampsia during the surgery, and myocardial infarction following the delivery. She was taking her antihypertensive medication as prescribed (aspirin 81 mg QD and nifedipine 30 mg BID) but admitted she failed to appear at her most recent prenatal visit one week prior. She was monitoring her blood pressure at home and had no concerns at the time. Before presenting to the emergency department, she experienced constant abdominal pain for two hours and no fetal movement for three hours. She has never smoked and does not drink or use illegal drugs.

**Figure 1:**
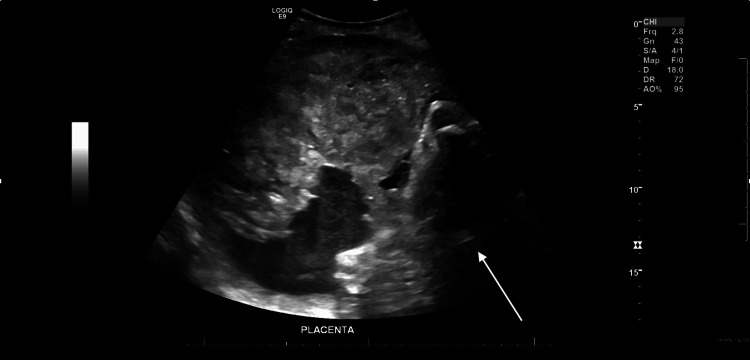
Ultrasonographic visualization demonstrating placental separation from the uterine wall (white arrow), consistent with abruption.

**Figure 2:**
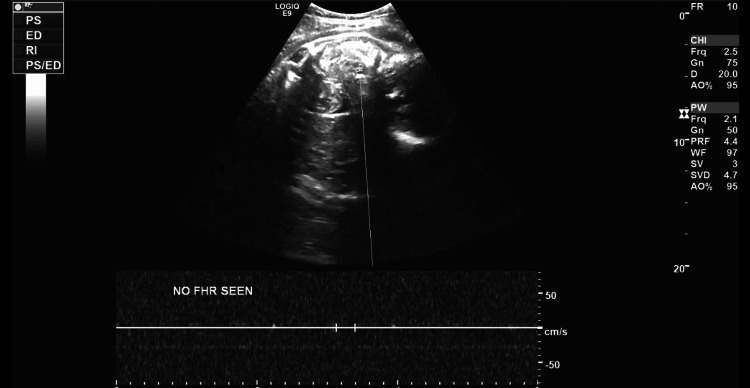
Ultrasonographic visualization demonstrating the absence of cardiac activity, confirming fetal demise.

Vital signs and examination revealed she was an afebrile patient with tachypnea, severe hypertension (BP 214/113 mmHg), and in obvious distress. An examination of the uterus showed a closed, long, and high cervix with tight contractions. No bleeding was noted. An emergency ultrasound showed an abnormal placenta with separation from the uterine wall (Figure 1), consistent with placental abruption, and verified the absence of fetal heart activity (Figure 2). Initial laboratory studies revealed hemoglobin 9.3 g/dL, hematocrit 29.6%, platelets 208×10³/μL, prothrombin time 16.8 seconds, partial thromboplastin time 28 seconds, and fibrinogen 107 mg/dL, indicating early coagulopathy (Table 1).

**Table 1:** Laboratory findings on admission. Abnormal values in bold suggest coagulopathy.

On Admission	Values	Reference
Hemoglobin	9.3 g/dL	12.0 - 15.5
Hematocrit	29.60%	36.1-44.3
Platelets	208x10^3/uL	150-450x10^3
Prothrombin	16.8 seconds	11-13.5
Partial Thromboplastin Time	28 seconds	25-35
Fibrinogen	107 mg/dL	200-400

**Figure 3:**
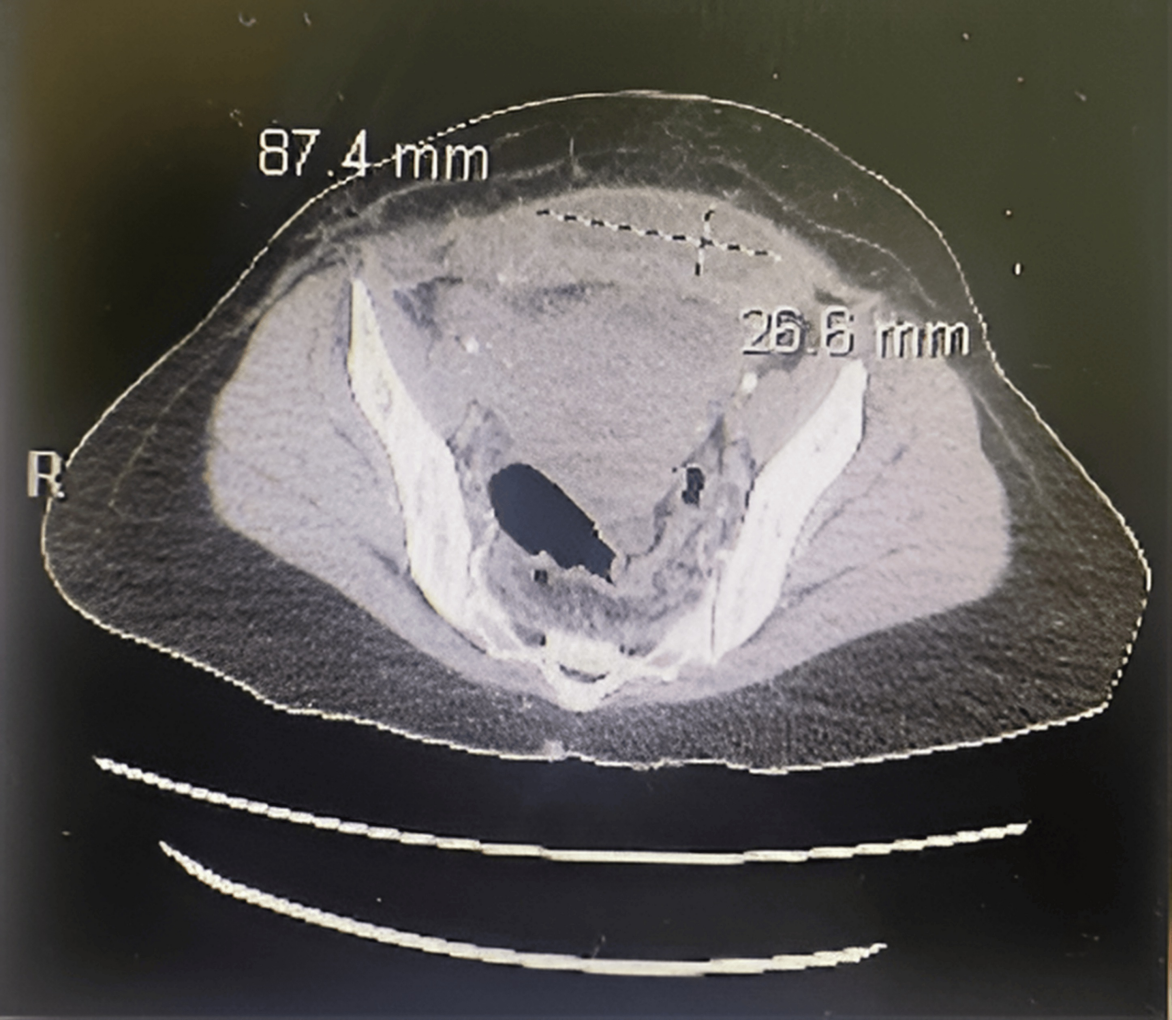
Computed tomography showing a complex hematoma measuring 87.4 x 26.6 mm, along the lower anterior abdominal wall and superior to the cesarean section scar.

Prior to the emergency cesarean delivery, the patient received four units of packed red blood cells (PRBCs) and one unit of fresh frozen plasma (FFP). One hour later, two more units of PRBCs, two units of FFP, and one unit of cryoprecipitate were administered intraoperatively. Laboratory tests conducted five hours after surgery showed creatinine 3.2 mg/dL, eGFR 15 mL/min/1.73 m^2^, and hemoglobin 6.9 g/dL, indicating acute kidney injury and worsening anemia (Table 2). Nephrology consultation diagnosed oliguric acute kidney injury superimposed on chronic kidney disease secondary to hypoperfusion and hemorrhage. She had increased bilateral cortical echogenicity on renal ultrasonography, which revealed underlying renal disease. She received stabilization and inpatient treatment for four days before being sent home. Later, she was diagnosed with a surgical site infection and complex hematoma (Figure 3), which were confirmed by CT imaging, when she returned to the emergency department a week later with bleeding from her surgical incision. She was treated with oral Augmentin 625 mg, taken every eight hours for seven days and monitored closely as an outpatient.

**Table 2:** Laboratory studies five hours after cesarean delivery. Abnormal values in bold suggest acute kidney injury and anemia.

5 Hours after Cesarean	Values	Reference
creatinine	3.2 mg/dL	0.5-1.1
eGFR	15 mL/min/1.73 m^2^	> 90
hemoglobin	6.9 g/dL	12.0 - 15.5

The patient has since recovered well and states she regrets her decision to miss her prenatal appointment. Soon after feeling the abdominal pain, she suspected a repeat abruption but had a difficult time coming to terms with the reality that this complication could befall her for a second time. 

## Discussion

Placental abruption has both acute and chronic forms clinically. Sudden onset vaginal bleeding is seen in the acute form, whereas recurrent episodes of mild to moderate hemorrhage over time are seen in the chronic form. The severity is primarily dependent on which maternal vessels are involved. Peripheral venous bleeding does not manifest as severe hemorrhage, yet it still needs to be closely monitored to prevent complications, while arterial bleeding that is centrally located is known to cause significant hemorrhage and immediate fetal compromise [6]. The triad of vaginal bleeding, abdominal pain, and uterine contractions is commonly seen in abruption, though uterine contractility is not a precise diagnostic indicator. The most frequent cause of bleeding is maternal decidual hemorrhage, but fetal vessels can occasionally be the source. The diagnosis is primarily clinical and requires ruling out alternative causes such as placenta previa or cervical changes brought on by active labor [2].

When placental abruption is clinically suspected, ultrasonography is the first imaging modality used to detect abnormal blood collections inside or next to placental tissue [7]. However, similar characteristics of placental tissue and blood products present diagnostic challenges, especially in advanced gestation. Investigations by Yeo and colleagues [8] recognized seven characteristic patterns associated with abruption: preplacental collections, "jello-like" undulation of the chorionic plate during fetal movement, retroplacental collections, marginal hemorrhage, subchorionic blood accumulation, placentomegaly with abnormal echogenicity, and intra-amniotic hematoma formation. Recognition of these patterns can help improve diagnostic accuracy, yet clinicians should remain aware that ultrasonography may still miss a substantial proportion of abruptions.

Risk factors

Abruption complicates 0.6% to 1.2% of pregnancies [3,4], and among the many risk factors, prior placental abruption is one of the most significant. Patients with a history of one prior abruption have a 10-fold increased risk of another, while two previous abruptions carry a remarkable 25-fold increased risk [5]. Similarly, the risk for abruption is increased in patients with chronic hypertension, which is 3- to 4-fold higher. Patients with preeclampsia face an increased risk of 4- to 6-fold, while there is an even greater danger in pregnancies with severe preeclamptic manifestations or hypertension compounded with pre-existing conditions [9-12].

Furthermore, maternal age exceeding 35 years, also known as advanced maternal age (AMA), is a growing trend in today’s socioeconomic environment and is quickly becoming another significant contributor to placental abruption risk [2]. Pregnancy in this population has an increased risk of maternal health challenges such as gestational diabetes, blood pressure abnormalities, and cesarean deliveries. There are associated fetal and neonatal adverse outcomes as well, such as genetic anomalies, spontaneous pregnancy loss, premature birth, postnatal intensive care requirements, and pregnancy loss [13-15]. Intrauterine fetal demise, especially when occurring beyond 20 weeks of gestation, shows a statistical association with advanced maternal age, although the exact relationship is under investigation. A comprehensive meta-analysis encompassing 44 studies and over 44 million births confirmed this association (OR 1.75, 95% CI 1.62-1.89) and proposed accelerated placental aging with progressive vascular dysfunction as the underlying mechanism [16].

Pathophysiology

The underlying pathophysiology of placental abruption involves interactions between acute events and chronic disease processes. For example, processes of thrombosis, inflammation, infection, and uteroplacental vasculopathy result in multiple vascular and structural abnormalities. These abnormalities lead to inadequate placental perfusion, compromised spiral artery development, areas of placental infarction, and insufficient trophoblastic penetration of maternal tissues. Then, in reaction to hemorrhage within the decidual layer, the resulting tissue hypoxia causes vascular endothelial growth factors to be upregulated, altering the body’s regular response to bleeding [2].

A major player involved in these processes is the hemostatic protein thrombin. Once decidual cells detect bleeding, they release tissue factor which initiates thrombin to begin the process of hemostasis [16,17]. This activation is responsible for upregulating vascular endothelial growth factors, which in turn stimulate further tissue factor expression and thrombin generation, creating a positive feedback loop that amplifies the hemostatic response and progressively impairs the body's ability to regulate bleeding. Simultaneously, this inflammatory cascade triggers increased breakdown of extracellular matrix through the expression of matrix metalloproteinases and pro-inflammatory cytokines such as interleukin-8 [17,18]. The resulting endothelial injury, combined with the dysregulated hemostatic processes, compromises the structural integrity of the uteroplacental interface. The combination of these pathophysiologic processes assists placental detachment and results in severe maternal hemorrhage.

Management

The management approach and treatment decisions for placental abruption are determined by three critical factors: the classification of abruption severity (ranging from Class 0 to Class 3), the current gestational age of the pregnancy, and whether there are signs of maternal compromise or fetal distress present. For patients diagnosed with Class 1 (mild) placental abruption who are carrying pregnancies less than 37 weeks gestation (preterm), conservative management may be appropriate when both maternal and fetal conditions remain stable [19]. This approach requires patients to follow intensified fetal surveillance procedures conducted once or twice weekly using non-stress testing and biophysical profile techniques [20] until 36 or 37 weeks when delivery is initiated [2]. Delivery will be delayed as long as possible to promote further fetal lung maturity and overall development, but only as long as both the mother and baby continue to show stable clinical parameters [19]. Management of chronic abruption cases such as these presents additional challenges, as current guidelines are limited [21] and data supporting specific protocols for either inpatient or outpatient care remains grossly insufficient.

In contrast, patients with Class 2 (moderate) or Class 3 (severe) placental abruption require immediate delivery if the fetus has reached viability. The mode of delivery depends on several clinical factors, with vaginal delivery being both feasible and advantageous when labor is progressing rapidly, and fetal monitoring shows no signs of compromise. This approach is particularly beneficial for patients who have developed coagulopathy, as it avoids the increased bleeding risks associated with surgical intervention. However, when fetal heart rate patterns indicate distress or when obstetric complications contraindicate vaginal delivery, emergency cesarean section becomes the required intervention to ensure optimal outcomes for both mother and child [19].

The initial assessment must include a comprehensive laboratory evaluation with coagulation profile and complete blood count, while arterial blood gas analysis, thromboelastography, and rotational thromboelastometry provide rapid results essential for transfusion management and resuscitation efforts. In cases of newly presenting vaginal hemorrhage at advanced gestational age, clinicians must assess severity through thorough history-taking and blood loss estimation. Since concealed bleeding can make hemorrhage difficult to quantify accurately, evaluation must also include assessment of vital signs, urine output, and mental status. Clinicians will commonly use the Advanced Trauma Life Support (ATLS) classification of hypovolemic shock as a general guide, but this system has significant limitations in pregnancy because it does not account for the unique physiological compensatory mechanisms that pregnant women possess and has not been validated for use in pregnancy. This limitation is particularly pronounced in patients with a history of hypertension or preeclampsia, where the ATLS system may underestimate the volume of fluids and blood products needed for proper resuscitation [2].

For high-risk pregnancies without diagnosed acute or chronic abruption, modifications to standard obstetric management protocols remain at the physician’s discretion, an approach that, as demonstrated in clinical practice, can lead to devastating consequences. These management gaps highlight several critical opportunities for improved clinical care and research aimed at preventing abruptions in high-risk populations. Essential improvements include: (i) The creation of evidence-based delivery and management protocols specifically designed for pregnancies at high risk of abruption; (ii) The development of comprehensive yet accessible patient education materials that address the compounding effects of multiple risk factors and emphasize recognition of warning signs requiring immediate medical attention; (iii) Healthcare systems should implement automated appointment reminder systems with escalation procedures specifically tailored for high-risk cases involving missed appointments; (iv) Lastly, specialized training should be provided for medical staff to recognize high-risk patients and identify warning signs that require immediate intervention and follow-up.

These systematic improvements represent crucial steps toward reducing the morbidity and mortality associated with placental abruption in vulnerable populations.

## Conclusions

In pregnancy, if the placenta separates from the uterine wall too soon, fetal blood supply becomes compromised, and there is significant maternal hemorrhage; this is known as placental abruption and is a serious obstetric emergency. Unfortunately, the available evidence supporting specific management protocols for inpatient and outpatient care is insufficient for both acute and chronic abruption cases. Even more concerning, pregnancies with an extremely high risk of abruption occurrence have no concrete management protocols.

This case illustrates this clinical challenge and is a prime example of placental abruption recurrence in patients with several compounding risk factors, including AMA, chronic hypertension during pregnancy, preeclampsia and eclampsia, and a history of a prior abruption. The fact that the patient missed a follow-up appointment the week prior to the abruption also emphasizes how clear communication and seamless collaboration between the patient and their medical team are essential to have the best chance for a healthy delivery. Nonetheless, what is most important for these high-risk pregnancies is the critical need for evidence-based monitoring guidelines. Simply put, further research is necessary to develop evidence-based recommendations for optimal management practices, delivery schedules, and risk stratification in this population as placental abruption continues to exist as a complex condition with high maternal and fetal morbidity and mortality, despite advancements in obstetric care.
